# Glucose in the hypothalamic paraventricular nucleus regulates GLP-1 release

**DOI:** 10.1172/jci.insight.132760

**Published:** 2020-03-31

**Authors:** Yue Ma, Risheka Ratnasabapathy, Ivan De Backer, Chioma Izzi-Engbeaya, Marie-Sophie Nguyen-Tu, Joyceline Cuenco, Ben Jones, Christopher D. John, Brian Y.H. Lam, Guy A. Rutter, Giles S.H. Yeo, Waljit S. Dhillo, James Gardiner

**Affiliations:** 1 Section of Endocrinology and Investigative Medicine and; 2 Section of Cell Biology and Functional Genomics, Department of Metabolism, Digestion and Reproduction, Imperial College London, London, United Kingdom.; 3 University of Cambridge Metabolic Research Laboratories and MRC Metabolic Diseases Unit, Wellcome Trust-MRC Institute of Metabolic Science, Addenbrooke’s Hospital, Cambridge, United Kingdom.

**Keywords:** Endocrinology, Neuroscience, Diabetes, Glucose metabolism, Peptides

## Abstract

Glucokinase (GK) is highly expressed in the hypothalamic paraventricular nucleus (PVN); however, its role is currently unknown. We found that GK in the PVN acts as part of a glucose-sensing mechanism within the PVN that regulates glucose homeostasis by controlling glucagon-like peptide 1 (GLP-1) release. GLP-1 is released from enteroendocrine L cells in response to oral glucose. Here we identify a brain mechanism critical to the release of GLP-1 in response to oral glucose. We show that increasing expression of GK or injection of glucose into the PVN increases GLP-1 release in response to oral glucose. On the contrary, decreasing expression of GK or injection of nonmetabolizable glucose into the PVN prevents GLP-1 release. Our results demonstrate that gluco-sensitive GK neurons in the PVN are critical to the response to oral glucose and subsequent release of GLP-1.

## Introduction

The hypothalamic paraventricular nucleus (PVN) is known to have roles in the regulation of energy homeostasis and is a key site for the regulation of the endocrine system. Neurons from the PVN project to several other regions of the brain, including the nucleus tractus solitarius (1, 2). From here second-order neurons project to peripheral organs, including the gastrointestinal tract (2). Glucokinase (GK) is an enzyme that has a critical role in glucose sensing (3, 4) and disposal (5, 6). GK within the central nervous system (CNS) is expressed in neurons, astrocytes, and tanycytes. In neurons it forms part of the glucose-sensing mechanism and is coexpressed with GLUT2 and ATP-sensitive potassium channels (K_ATP_) (7). GK is expressed in numerous hypothalamic nuclei, including the ventromedial nucleus (VMN), arcuate nucleus (ARC), and PVN (8, 9). Within the VMN, GK has been established as being an important regulator of the counterregulatory response to hypoglycemia (10–12). GK within the ARC regulates food intake and glucose homeostasis (13–16). Given the sparsity of data on the PVN’s role in glucose homeostasis and because GK is expressed in the PVN, and this nucleus is known to have extensive neuronal connections to the pancreas (16–18), we sought to investigate the role of glucose sensing in the PVN in glucose homeostasis.

## Results

### Increased GK activity within the PVN improves glucose homeostasis.

**Figure 1:**
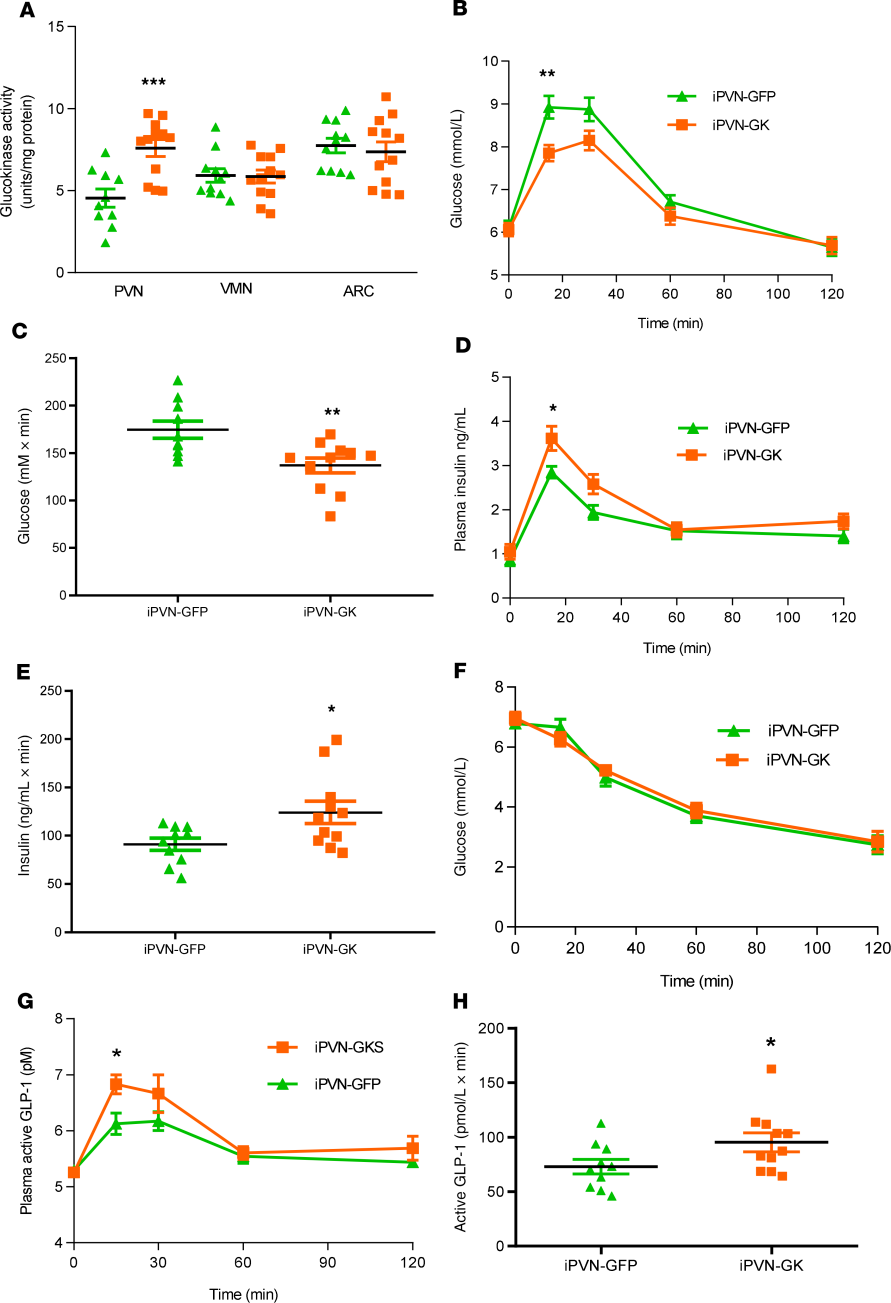
Effect of genetically increased PVN GK activity on oral glucose tolerance test. Groups of adult male Wistar rats were injected with either rAAV-GFP (iPVN-GFP) or rAAV-GK (iPVN-GK) bilaterally into the PVN. They then underwent an oral glucose tolerance test (oGTT) and an insulin tolerance test (ITT). (**A**) GK activity in ARC, VMN, and PVN of iPVN-GFP (shown in green) or iPVN-GK (shown in orange) rats. (**B**) Plasma glucose during an oGTT. (**C**) Incremental AUC (iAUC) of 0- to 60-minute plasma glucose during an oGTT. (**D**) Plasma insulin levels during an oGTT. (**E**) iAUC of 0- to 60-minute plasma insulin during an oGTT. (**F**) Plasma glucose levels during an ITT. (**G**) Plasma GLP-1 levels during an oGTT. (**H**) iAUC of 0- to 60-minute plasma GLP-1 during an oGTT. Data are represented as mean ± SEM; *n* = 10 (GFP) and 11 (GK). *P < 0.05, **P < 0.01, and ***P < 0.001. Data for **A** were analyzed by ANOVA and post hoc Holm-Šídák; data for **B**, **D**, **F**, and **G** were analyzed by 2-way ANOVA and post hoc Holm-Šídák; data for **C**, **E**, and **H** were analyzed by *t* test.

Rats were injected with previously validated recombinant adeno-associated virus 2 (rAAV-2) (13, 14) encoding GK into the PVN (intra-PVN GK, iPVN-GK) or rAAV encoding GFP as a control (iPVN-GFP). As we and others have previously shown, expression was limited to neurons and did not occur in glial cells (14, 19) (Supplemental Figure 1; supplemental material available online with this article; https://doi.org/10.1172/jci.insight.132760DS1). GK activity was increased by 40% in iPVN-GK compared with iPVN-GFP animals. As expected, there was no difference in GK activity in either the ARC or VMN (Figure 1A). During an oGTT, iPVN-GK rats had significantly lower plasma glucose levels than iPVN-GFP rats at 15 minutes (Figure 1, B and C). Insulin levels were increased at 15 minutes in iPVN-GK compared with iPVN-GFP animals (Figure 1, D and E). However, insulin sensitivity was unchanged as demonstrated by an ITT (Figure 1F). Body weight gain and food intake were unchanged (Supplemental Figure 2, A and B). These data suggested that PVN GK regulates glucose-stimulated insulin secretion. We therefore sought to investigate potential mechanisms of action.

### Increased PVN GK activity increases glucagon-like peptide-1 release.

In light of the increased glucose-stimulated insulin release, we measured active glucagon-like peptide 1 (GLP-1) levels during oGTT. Plasma GLP-1 levels were significantly higher in iPVN-GK than in iPVN-GFP rats at 15 minutes (Figure 1, G and H). Levels of active GLP-1 in controls were in line with those reported elsewhere (20–22).

### Decreased GK activity within the PVN impairs glucose tolerance and blunts GLP-1 release.

**Figure 2:**
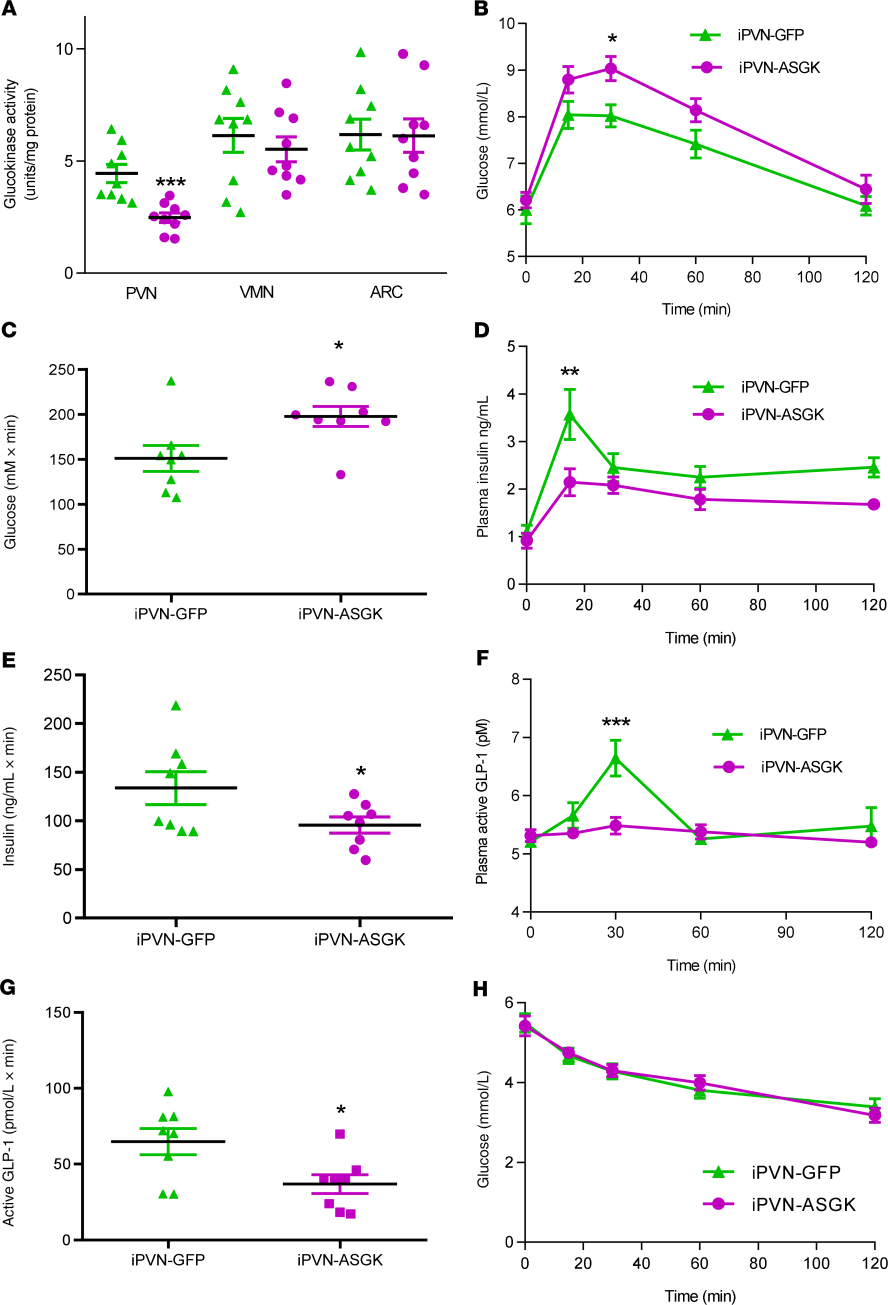
Effect of genetically decreased PVN GK activity on oGTT. Groups of adult male Wistar rats were injected with either rAAV-GFP (iPVN-GFP) or rAAV-ASGK (iPVN-ASGK) bilaterally into the PVN. They then underwent an oGTT and ITT. (**A**) GK activity in ARC, VMN, and PVN of iPVN-GFP (green) or iPVN-ASGK (magenta). (**B**) Plasma glucose during an oGTT in iPVN-GFP and iPVN-ASGK rats. (**C**) AUC analysis of 0- to 60-minute plasma glucose during an oGTT in iPVN-GFP and iPVN-ASGK rats. (**D**) Plasma insulin levels during an oGTT in iPVN-GFP and iPVN-ASGK rats. (**E**) AUC analysis of 0- to 60-minute plasma insulin during an oGTT in iPVN-GFP and iPVN-ASGK rats. (**F**) Plasma GLP-1 levels during an oGTT in iPVN-GFP and iPVN-ASGK rats. (**G**) AUC analysis of 0- to 60-minute plasma GLP-1 during an oGTT in iPVN-GFP (triangles) and iPVN-ASGK (squares) rats. (**H**) Plasma glucose levels during an ITT in iPVN-GFP and iPVN-ASGK rats. Data are represented as mean ± SEM; *n* = 8. *P < 0.05, **P < 0.01, ***P < 0.001. Data for **A** were analyzed by 1-way ANOVA and post hoc Holm-Šídák; data for **B**, **D**, **F**, and **G** were analyzed by 2-way ANOVA and post hoc Holm-Šídák; data for **C**, **E**, and **H** were analyzed by *t* test.

It is possible that the effect on GLP-1 release was due to pharmacological effects of overexpression of GK in the PVN. We therefore sought to investigate the physiological effect of PVN GK. To do this, we decreased GK activity within the PVN using bilateral injection of previously validated rAAV-2 (13, 14) encoding antisense GK (iPVN-ASGK). GK activity in the PVN was decreased by 45% in iPVN-ASGK rats compared with iPVN-GFP rats (Figure 2A). As expected, GK activity in the VMN and ARC was unaffected (Figure 2A). During an oGTT, plasma glucose levels at 30 minutes were significantly higher in iPVN-ASGK compared with iPVN-GFP rats (Figure 2, B and C). In keeping with this, plasma insulin levels were significantly reduced 15 minutes after glucose ingestion in iPVN-ASGK compared with iARC-GFP rats (Figure 2, D and E). Importantly, the GLP-1 response to oral glucose was absent in iARC-ASGK rats (Figure 2, F and G). Similar to the effects of GK overexpression, insulin sensitivity was not altered (Figure 2H). Body weight gain and food intake were unchanged (Supplemental Figure 3, A and B).

This suggests that GK in the PVN is physiologically regulating the release of GLP-1 in response to oral glucose. However, it is possible that the changes in GLP-1 levels were secondary to other changes in glucose homeostasis or insulin release.

### Altered PVN GK activity does not regulate glucose homeostasis during intraperitoneal GTT.

We therefore sought to establish whether altered PVN GK activity affected glucose homeostasis in the absence of the GLP-1 response. Thus, intraperitoneal GTTs (ipGTTs) were performed in rats with altered PVN GK activity because during ipGTT plasma GLP-1 levels are unaffected. Plasma glucose, plasma insulin, and plasma GLP-1 levels were not changed in either iPVN-GK rats compared with controls (Supplemental Figure 2, C–E) or iPVN-ASGK rats compared with controls (Supplemental Figure 3, C–E). This suggests that the effects of altered PVN GK on glucose homeostasis are dependent upon changes in GLP-1 release.

### Intravenous injection of glucose in conjunction with increased PVN GK activity does not release GLP-1.

**Figure 3:**
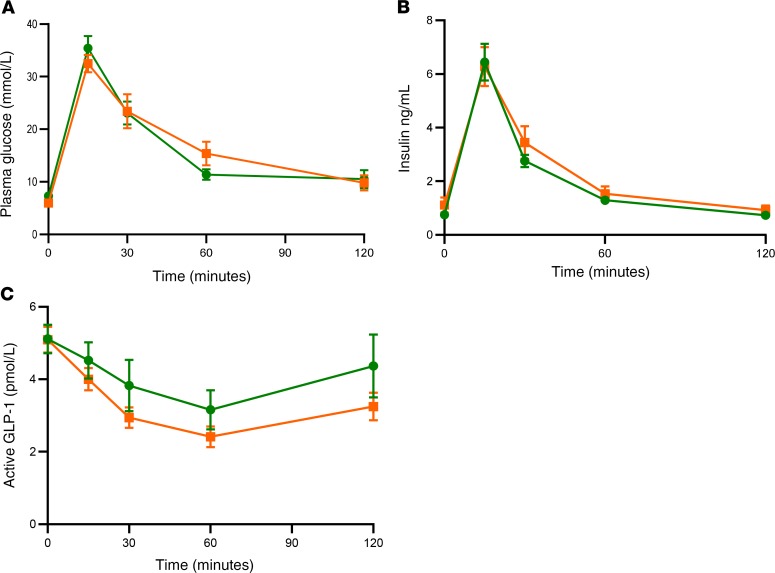
Effect of increased PVN GK activity during an ivGTT. Groups of adult male Wistar rats were injected with either vehicle (green circles) or 0.5 nmol CpdA (orange squares) into the PVN. They were then subjected to an ivGTT. (**A**) Plasma glucose during an ivGTT in vehicle-injected (green circles) and CpdA-injected (orange squares) rat. (**B**) Plasma insulin during an ivGTT in vehicle-injected (green circles) and CpdA-injected (orange squares) rats. (**C**) Plasma active GLP-1 during an ivGTT in vehicle-injected (green circles) and CpdA-injected (orange squares) rats. Data are shown as mean ± SEM; *n* = 9. Data were analyzed by 2-way ANOVA and post hoc Holm-Šídák.

To further confirm that oral glucose delivery was required to regulate GLP-1 release when PVN GK activity was increased, we performed an intravenous GTT (ivGTT) while concurrently injecting Compound A (CpdA), a specific GK activator, into the PVN of male Wistar rats. Injection of CpdA during an ivGTT did not affect plasma insulin, glucose, or GLP-1 levels (Figure 3, A–C).

### Acute increases in GK activity within the PVN regulate GLP-1 release.

**Figure 4:**
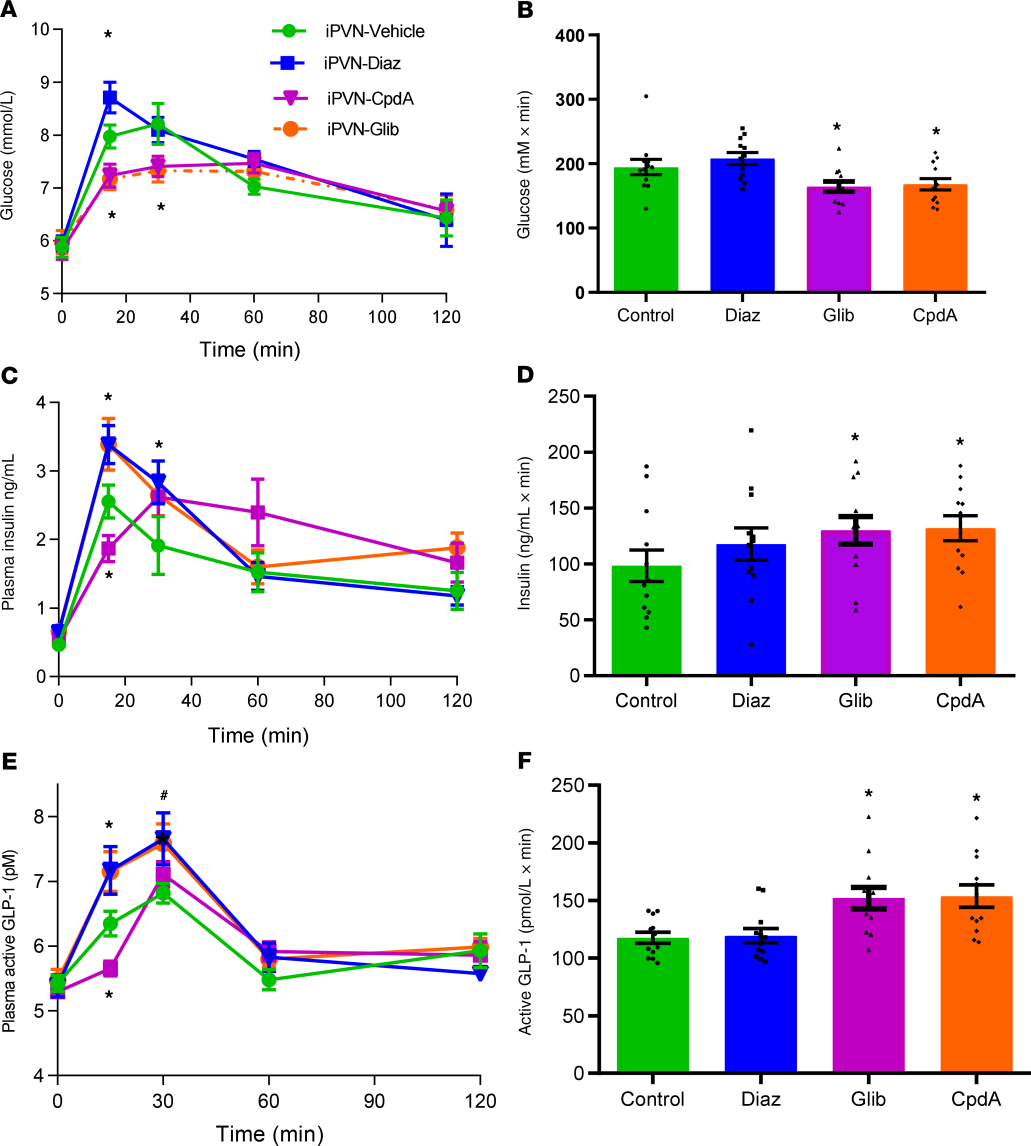
Effect of pharmacologically increased paraventricular GK activity and manipulating K_ATP_ activity on glucose homeostasis in Wistar rats. Adult male Wistar rats were injected into the PVN with either vehicle (veh) or 0.5 nmol of the GK activator Compound A (CpdA), 2 nmol glibenclamide (Glib), or 1 nmol diazoxide (Diaz). (**A**) Plasma glucose during an oGTT. (**B**) iAUC of 0- to 60-minute plasma glucose during an oGTT. (**C**) Plasma insulin levels during an oGTT. (**D**) iAUC of 0- to 60-minute plasma insulin during an oGTT. (**E**) Plasma GLP-1 during an oGTT. (**F**) iAUC of 0- to 60-minute plasma GLP-1 during an oGTT. Data are represented as mean ± SEM; *n* = 12. *P < 0.05 vs. control. Data for **A**, **C**, and **E** were analyzed by 2-way ANOVA and post hoc Holm-Šídák; data for **B**, **D**, and **F** were analyzed by 1-way ANOVA and post hoc Holm-Šídák.

Using rAAV to alter GK expression results in long-term changes in GK activity. To investigate whether these long-term changes in GK activity in the PVN were necessary to alter GLP-1 levels during an oGTT, we investigated the impact of acute changes in PVN GK activity. CpdA reduced plasma glucose levels and increased insulin levels during an oGTT (Figure 4, A–D). Importantly, this acute activation of GK also increased GLP-1 levels during an oGTT in the same fashion as observed with long-term increases in GK activity (Figure 4, E and F). This suggests that long-term changes in GK activity are not required to alter GLP-1 release during an oGTT. This implies that the changes in GLP-1 response are not due to long-term changes in innervation of the L cells or of neuronal connectivity in the PVN.

### Antagonizing the GLP-1 receptor reduces the effect of increased PVN GK activity.

**Figure 5:**
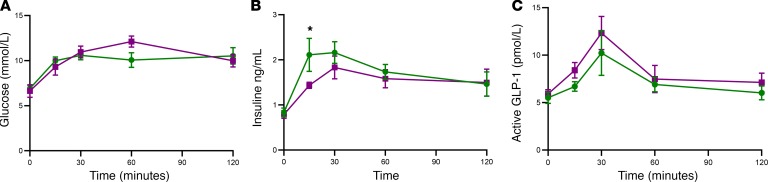
Effect of administration of the GLP-1 antagonist exendin(9-39) during an oGTT with coadministration of iPVN CpdA. Groups of adult male Wistar rats were injected intraperitoneally with either vehicle (green circles) or 100 μg/kg of exendin(9-39) (magenta squares). Next, 0.5 nmol of CpdA was injected into the PVN. They were then subjected to an oGTT. (**A**) Plasma glucose during an oGTT in vehicle-injected (green circles) and exendin-injected (magenta squares) rats. (**B**) Plasma insulin during an oGTT in vehicle-injected (green circles) and exendin(9-39)–injected (magenta squares) rats. (**C**) Plasma active GLP-1 during an ivGTT in vehicle-injected iPVN-GFP (green circles) and CpdA-injected (magenta squares) rats. Data are shown as mean ± SEM; *n* = 10. Data were analyzed by 2-way ANOVA and post hoc Holm-Šídák. *P < 0.05.

We sought to confirm that changes in glucose homeostasis during an oGTT following iPVN GK activation were due to increased GLP-1 release. Thus we administered the GLP-1 receptor antagonist exendin(9-39) during an oGTT while concurrently administering CpdA into the PVN. Concurrent administration of exendin(9-39) reduced the effect of iPVN CpdA on insulin and glucose levels (Figure 5).

### PVN K_ATP_ activity regulates GLP-1 release.

GK is known to signal via closure of the K_ATP_ to mediate its effects in pancreatic β cells and the hypothalamus (4, 7, 13, 14). To test if this was also plausible in the regulation of GLP-1 release, we administered the K_ATP_ blocker glibenclamide or the K_ATP_ activator diazoxide into the PVN of male Wistar rats. iPVN glibenclamide decreased plasma glucose levels during an oGTT while increasing insulin and GLP-1 levels (Figure 4), mimicking the effects of increased GK activity. In contrast, iPVN diazoxide increased glucose levels while decreasing plasma insulin and GLP-1 levels (Figure 4). These results suggest that GK may be acting via K_ATP_ in the PVN to regulate GLP-1 release and that signals generated in the PVN regulate GLP-1 release.

### GLP-1 release requires glucose metabolism in the PVN.

**Figure 6:**
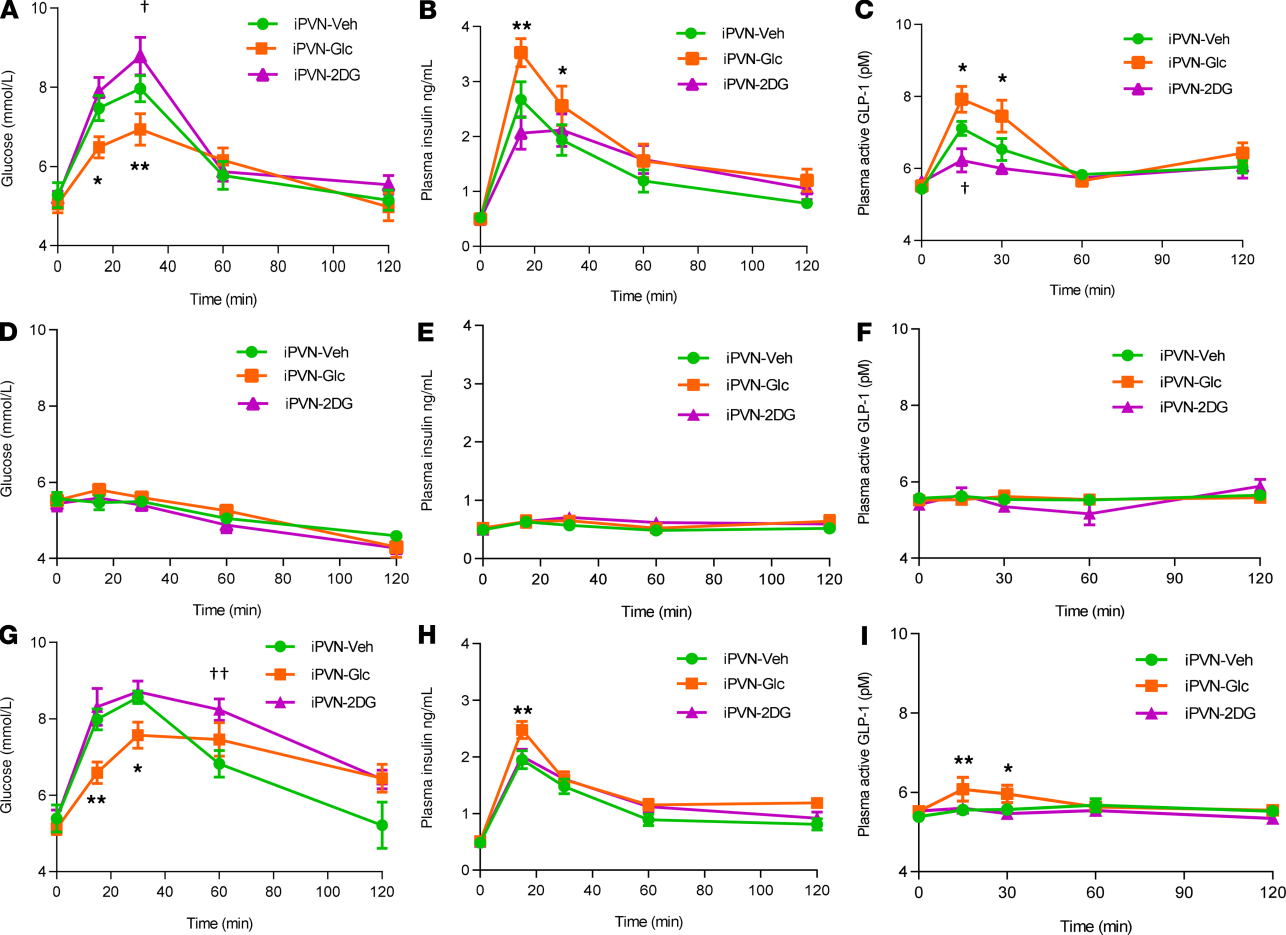
Effect of pharmacological administration of D-glucose and 2-deoxy-d-glucose versus vehicle administration on glucose homeostasis in Wistar rats. Adult male Wistar rats were injected into the PVN with either vehicle (Veh) or 250 μg of 2-deoxy-d-glucose (2DG) or 1.5 μg D-glucose (Glc). (**A**) Plasma glucose during an oGTT. (**B**) Plasma insulin levels during an oGTT. (**C**) Plasma GLP-1 during an oGTT. (**D**) Plasma glucose where no glucose is given orally or IP. (**E**) Plasma insulin levels where no glucose is given orally or IP. (**F**) Plasma GLP-1 where no glucose is given orally or IP. (**G**) Plasma glucose during an ipGTT. (**H**) Plasma insulin during an ipGTT. (**I**) Plasma GLP-1 levels during an ipGTT. Data are represented as mean ± SEM; *n* = 10. *P < 0.05, and **P < 0.01 D-glucose vs. vehicle; †*P* < 0.05, and ††*P* < 0.01 2DG vs. vehicle. Data were analyzed by 2-way ANOVA with post hoc Holm-Šídák.

We next sought to determine whether the release of GLP-1 is dependent upon the presence of glucose, and its subsequent metabolism in the PVN, or whether GK was acting independently of this. We administered vehicle, glucose, or 2-deoxy-d-glucose (2-DG), a competitive inhibitor of GK, directly into the PVN. During a subsequent oGTT, iPVN glucose decreased plasma glucose levels and increased plasma insulin levels compared with controls (Figure 6, A and B). iPVN glucose also increased plasma GLP-1 levels during the oGTT (Figure 6C), suggesting that entry of glucose into the PVN may be a limiting factor in GLP-1 release in response to oral glucose. iPVN 2-DG resulted in failure of oral glucose to induce GLP-1 release (Figure 6C), and this was accompanied by increased glucose levels and reduced insulin levels (Figure 6, A and B).

### Glucose injection into the PVN alone, with intraperitoneal glucose injection, does not release GLP-1.

Release of GLP-1 occurs after ingestion of glucose and not following intraperitoneal (IP) or other peripheral injections of glucose (23). However, following IP injection, glucose takes up to 20 minutes to enter the brain (24). We therefore investigated whether the lack of GLP-1 release following IP administration of glucose was due to delayed glucose entry into the brain. Plasma GLP-1, glucose, and insulin levels were unchanged following injection of either glucose or 2-DG (Figure 6, D–F) in fasted rats, demonstrating that the presence of glucose in the PVN in isolation is insufficient to elicit GLP-1 release.

iPVN glucose during an ipGTT did reduce plasma glucose levels while iPVN 2-DG increased plasma glucose levels (Figure 6G). Interestingly, the pattern of changes was different from that seen during an oGTT. Thus, following iPVN 2-DG, glucose levels were increased at 60 minutes during the ipGTT while levels at other time points were unchanged (Figure 6G). However, during the oGTT, glucose levels were increased at 30 minutes with other time points unchanged (Figure 6A). iPVN glucose produced a very small but nonetheless significant increase in insulin levels at 15 minutes but not at other times (Figure 6H). iPVN 2-DG did not affect plasma insulin (Figure 6H). iPVN glucose during an ipGTT did result in a minimal but statistically significant rise in plasma GLP-1 (Figure 6I). However, this was not of the same order of release as during either an oGTT or when with iPVN glucose during an oGTT (Figure 6C).

## Discussion

GK is highly expressed in the PVN, but its role within this nucleus is unknown. We therefore investigated the role of PVN GK using our previously validated rAAV-2 to increase GK activity specifically in the PVN. When we performed an oGTT in these animals, we found improved glucose tolerance with reduced glucose excursion and increased insulin release. This suggested that elevated PVN GK enhances insulin release. We therefore sought to investigate the mechanism(s) by which PVN GK was regulating insulin release. Interestingly, we found that increased PVN GK activity also increased GLP-1 release during an oGTT. Further supporting the physiological relevance of this finding, decreasing GK activity in the PVN reduced GLP-1 release in response to an oral glucose load.

The phenotype of the neurons expressing GK in the PVN is not yet known, and it is likely that there are a variety of neurons expressing GK in the PVN. Moreover these may innervate multiple targets; it is known that the PVN has connections to multiple organs, including the gastrointestinal tract (2) and the pancreas (3–5). It is therefore possible that PVN GK regulates insulin release and therefore glucose homeostasis by mechanisms other than the modulation of GLP-1 release. Indeed, the data from the iPVN administration of glucose and 2-DG during an ipGTT suggest this is possible. However, when we administered the GLP-1 receptor antagonist exendin(9-39), this reduced the changes in glucose homeostasis previously seen. This suggests that any direct effects on insulin release are likely to be a minor contributor to the regulation of glucose homeostasis by PVN GK. Nonetheless, it is possible that under different physiological conditions PVN neurons expressing GK may regulate glucose homeostasis by direct regulation of insulin release.

It is possible that PVN glucose detection regulates GLP-1 release indirectly. One possibility is that altered GLP-1 release is secondary to changes in gut motility because the PVN is known to regulate gut motility (25). However, this explanation seems unlikely because plasma glucose increases at the same rate during glucose tolerance tests irrespective of PVN GK activity or iPVN glucose injection. Indeed, if changes in gut motility do explain these data, one would expect the changes in glucose levels to be slowest in conditions where GLP-1 did not change, and this was not the case. The data suggest that any changes in gut motility are likely to be a minor component of the effect on GLP-1 release. Another possible explanation for the changes in GLP-1 levels is that they reflect alterations in dipeptidyl peptidase-4 (DPPIV) activity (26). However, this seems to be an unlikely mechanism because DPPIV is expressed widely within the body and is present in the blood, so a mechanism regulating its activity would be difficult to envisage. It is also possible that the effects we have observed following 2-DG administration are indirect and are due to local glucoprivic effects. Though possible, this seems unlikely given the similar results produced from different experimental approaches and the opposite effects produced by injection of glucose.

Our data therefore suggest a potentially new mechanism that regulates the release of GLP-1 in response to oral glucose. It is well known that mechanisms intrinsic to L cells regulate GLP-1 release (23, 27, 28). However, there is also evidence of neuronal regulation of GLP-1 secretion (29–33). The PVN innervates the gastrointestinal tract via both sympathetic and parasympathetic efferent signals. Our data show that detection of glucose in the PVN alone or in concert with increased circulating levels is insufficient to stimulate GLP-1 release. It is therefore possible that PVN glucose sensing alters the intrinsic sensitivity of L cells to plasma glucose, altering the GLP-1 response to a glucose load.

Perhaps the more likely interpretation of our data is that PVN glucose sensing serves a permissive role and removes an inhibitory input into L cells, which prevents GLP-1 release. An inhibitory sympathetic input into L cells has been demonstrated in pigs (29). It is therefore possible that this is regulated by glucose detection in the PVN. PVN GK neurons having an inhibitory input would explain why a 60% reduction in PVN GK activity and the injection of 2-DG into the PVN prevented the release of GLP-1 completely. An inhibitory signal to L cells would also explain why in vitro L cell preparations release GLP-1 in a glucose-responsive fashion because it would not be active in these circumstances. Other data support this interpretation. Thus, glucose injection into the PVN was not sufficient to elicit GLP-1 release on its own or in the presence of increased circulating glucose. Moreover, elevated GK expression in the PVN does not result in GLP-1 release during an ipGTT or during an ivGTT. Together, this suggests that the release of GLP-1 is dependent upon delivery of glucose to the lumen of the gastrointestinal tract in combination with delivery to the PVN. However, from our data it is not yet possible to determine the relative contribution of each of these systems in the regulation of GLP-1 release.

It is plausible that the above systems act to protect the brain from hypoglycemia during starvation particularly upon refeeding. The brain is dependent upon glucose to maintain proper function. During periods of starvation when brain glucose levels are low, PVN GK would be inactivated and the inhibitory signal would be maintained. Thus, when food is consumed the incretin response would be inhibited, allowing the glucose levels to be maintained at a higher level. Once normoglycemia had been achieved, the PVN GK would be activated and the normal incretin response, including increased insulin secretion, would occur if glucose were still in the gastrointestinal tract. This system could be particularly important during periods of starvation where meals contain little glucose but large amounts of fat and protein, which also stimulate release of GLP-1. Under these conditions, the system would protect the brain from potentially damaging hypoglycemia by preventing the release of GLP-1 and subsequently insulin.

In summary, our observations demonstrate that glucose sensing in a GK-dependent fashion within the PVN is required for the normal release of GLP-1. It is well established that the brain regulates glucose homeostasis by several mechanisms, including changes in insulin secretion, the response to hypoglycemia, and glucose handling in the liver (10, 34, 35). This obligatory system requiring input from both the brain and periphery has not previously been hypothesized to our knowledge. However, it is likely that many similar and, as yet, undiscovered systems of dual control between the CNS and peripheral organs exist, which regulate a range of physiological systems and pathological responses.

## Methods

### rAAV production.

Full-length GK cDNA was isolated and amplified by PCR from the plasmid pCMV4.GKB1 encoding full-length GK cDNA (gift from Mark Magnuson, Vanderbilt University Medical Center, Nashville, Tennessee, USA). In order to construct the GK sense (sGK) and ASGK, GK DNA underwent subcloning in the forward (GKS) and reverse (GKAS) orientation into the plasmid pTR-CGW (gift from Joost Verhaagen, Nederlands Herseninstituut, Amsterdam, Netherlands). We used helper plasmid pDG to produce rAAV particles in a 2-plasmid system (36). The rAAV particles were then recovered and purified using an iodixanol gradient to make rAAV sense GK (rAAV-GK) and rAAV antisense GK (rAAV-ASGK)(37).

### Animal experiments.

Adult male Wistar rats (230–280 g, Charles River UK Ltd) were individually housed and maintained in a controlled environment (temperature 21°C–23°C, 12-hour light/12-hour dark cycle, lights on at 0700 hours). They had ad libitum access to standard chow (RM1 diet; Special Diet Services UK Ltd) and water except where stated.

### Bilateral iPVN rAAV microinjection.

Half a microliter of either rAAV-GK (titer: 2.96 × 10^12^ genome particles/mL) or rAAV-ASGK (titer: 3.42 × 10^12^ genome particles/mL) or GFP (titer: 5.04 ×10^12^ genome particles/mL) as a control was bilaterally injected into the PVN of male Wistar rats using stereotactic surgery. Coordinates were determined from Paxinos Watson (38) and were 1.8 mm posterior to bregma, 0.5 mm lateral to bregma, and 8 mm below the skull surface.

The stereotactic surgery was carried out as previously described (13). Male Wistar rats were anesthetized with oxygen (2 L/min) and 4% isoflurane and held in a stereotactic frame. The surgical area was shaved, then cleaned with betadine. A rostrocaudal incision (approximately 1 cm) was made in the skin over the vertex of the skull, and the periosteum from the underlying bone was removed to expose the bregma. Bilateral burr holes were drilled using an electric drill according to coordinates calculated previously. Each animal received an iPVN injection of 0.5 μL rAAV into both sides at a rate of 12 μL/h over 5 minutes using a stainless steel injector and an infusion pump. The cannula and injector were left in position for 5 minutes to limit back diffusion and then slowly withdrawn. The scalp incision was sutured with a 4.0 polypropylene suture (Ethicon).

### Intraperiventricular administration of pharmacological agents.

Animals were prepared and mounted onto a stereotactic frame as described previously. Anesthetic, analgesia, and antibiotics were administered as previously described (13). A unilateral burr hole was drilled using an electric drill according to coordinates listed previously (1.8 mm posterior to bregma, 0.5 mm lateral to bregma, and 8 mm below the skull surface). A stainless steel guide cannula held by an arm mounted onto the stereotactic frame was inserted 7.5 mm below the skull surface. Three stainless steel screws were then gently inserted into the skull using a hand drill to act as anchors. Dental acrylic was used to form a pedestal fixing the cannula into a stationary position with support from the mounting screws. This was left to dry and a dummy cap was screwed onto the cannula to prevent infection.

Subsequently rats were fasted overnight and the following morning injected into the PVN via a stainless steel cannula that projected 0.5 mm beyond the guide cannula with one of the following in a volume of 0.5 μL: saline, 0.5 nmol CpdA (a GK activator) — 2-Amino-5-(4-methyl-4H-(1,2,4)-triazole-3-yl-sulfanyl)-*N*-(4-methyl-thiazole-2-yl)benzamide (CAS 603108-44-7, MilliporeSigma) — 1 nmol diazoxide (a K_ATP_ activator), or 2 nmol glibenclamide (a K_ATP_ blocker). These doses are identical to those used previously (13). Thirty minutes after the injection the animals underwent an oGTT as described below. The experiment was a crossover design with each animal receiving each injection, in a random order, at least 3 days apart.

In another study, animals were injected with one of the following in a volume of 0.5 μL: saline, 1.5 μg D-glucose (39), or 0.25 mg 2-DG (40), then underwent an oGTT, ipGTT, or ivGTT as described below.

At the end of study, 1 μL of India ink was injected through the cannula and the brains were dissected out. Cresyl violet staining was performed on brain slices to determine the accuracy of the cannulation; all cannulae were correctly targeted to the PVN.

### Oral glucose tolerance tests.

All rats underwent an oGTT 4 weeks postsurgery. Animals were fasted overnight prior to the study. A 24-gauge/19-mm cannula was inserted into the tail vein. The baseline blood sample was collected at 0 minutes. A 2.5 g/kg dose of glucose (20% dextrose diluted in glass distilled water, GDW) was then administered orally to each animal. Following glucose consumption, blood was taken at 15, 30, 60, and 120 minutes into lithium heparin microvettes with DPPIV inhibitor added to a final concentration of 50 μM. Heparin was administered to each sample to prevent clotting. Plasma was separated from blood cells by centrifugation at 13,000 *g* for 5 minutes at 4°C and was stored at –80°C.

### Intraperitoneal glucose tolerance tests.

For ipGTTs, animals were fasted overnight prior to the study. A 24-gauge/19-mm cannula was inserted into the tail vein. The baseline blood sample was collected at 0 minutes. A 2.5 g/kg dose of glucose (20% dextrose diluted in GDW) was then administered by IP injection. Following glucose injection, blood was taken at 15, 30, 60, and 120 minutes into lithium heparin microvettes with DPPIV inhibitor added to a final concentration of 50 μM. Heparin was administered after each sample to prevent clotting. Plasma was separated from blood cells by centrifugation at 13,000 *g* for 5 minutes at 4°C and was stored at –80°C.

### Intravenous glucose tolerance tests.

For ivGTTs, animals were fasted overnight prior to the study. A 24-gauge/19-mm cannula was inserted into the tail vein. The baseline blood sample was collected at 0 minutes. A 2 g/kg dose of glucose (30% w/v) was administered via catheter and followed by 200-μL saline flush. Following glucose injection, blood was taken at 15, 30, 60, and 120 minutes into lithium heparin microvettes with DPPIV inhibitor added to a final concentration of 50 μM. Heparin was administered after each sample to prevent clotting. Plasma was separated from blood cells by centrifugation at 13,000 *g* for 5 minutes at 4°C and was stored at –80°C.

### ITTs.

All rats underwent an ITT 5 weeks after PVN surgery. A 24-gauge/19-mm cannula was inserted into the tail vein. The baseline blood sample was collected at 0 minutes. A 2 U/kg dose of insulin was injected IP. Blood was taken at 15, 30, 60, and 120 minutes. Plasma was separated by centrifugation at 13,000 *g* for 5 minutes at 4°C and was stored at –80°C.

### Collection of tissue samples.

Unless otherwise stated in Methods, animals from all studies were euthanized in early light phase. Pancreas, ileum, and brain were collected from all animals following the completion of a study.

### GK activity assay in isolated PVN, VMN, and ARC samples.

The GK activity in the hypothalamic nuclei was measured as previously described (13). In brief, 300-μm-thick coronal sections of brain tissue were cut using a cryostat with a chamber temperature of –7°C and transferred to glass slides. Slides were kept on dry ice to keep them frozen, and the PVN, ARC, and VMN were collected using a 22-gauge neuro punch (Fine Science Tools) using a rat brain atlas as a guide (38). Nuclei were homogenized in 200 μL extraction buffer containing 0.0107 M MgCl_2_, 5 mM sodium EDTA, 0.15 M KCl, and 0.07% w/v 2-mercaptoethanol. Fifty microliters of each supernatant was added to 500 μL reaction mix containing 100 mM Gly-Gly, 45 μM 5-thio-d-glucose-6-phosphate, 1 M MgCl_2_, 0.5 mM 3-O-methyl-*N*-acetylglucosamine, 200 mM ATP, 12.5 mM NADP, 2 M glucose, and 0.4 IU GK (type IX, from Baker’s yeast) and incubated at 37°C for 30 minutes. Each sample was set up in triplicate. Absorbance of each sample was read at 340 nm, and GK concentration was calculated by extrapolating values from the standard curve for GK. Samples were normalized to protein content using a Pierce bicinchoninic acid assay (Thermo Fisher Scientific).

### Measurement of glucose in plasma samples using a glucose oxidase assay.

Plasma glucose levels were measured using a glucose oxidase assay (Randox) according to the manufacturer’s instructions and the absorbance read using ELx808 Microplate Reader (Biotek Instruments Ltd).

### Measurement of insulin in plasma samples using ELISA.

Plasma insulin levels were analyzed using ultrasensitive rat insulin ELISA kit from Crystal Chem as per manufacturer’s instructions. Microplates were read by ELx808 Microplate Reader.

### Measurement of active GLP-1 in plasma samples using ELISA.

Plasma active GLP-1 levels were measured using GLP-1 (Active) ELISA from MilliporeSigma as per manufacturer’s instructions, including the use of DPP4 inhibitor during sample collection. The microplates were read by SpectraMax i3x Platform (Molecular Devices).

### Quantitative reverse transcription PCR.

Frozen tissues were homogenized by TissueLyser II (QIAGEN) in TRIzol (Invitrogen, Thermo Fisher Scientific), and total RNAs were isolated by the RNeasy Mini Kit with on-column DNase treatment (QIAGEN). cDNA was synthesized by High-Capacity cDNA Reverse Transcription Kit (Thermo Fisher Scientific). Quantitative PCR was conducted using TaqMan Gene Expression Assays (primer assay IDs ins1 Rn02121433_g1, Gcg Rn00562293_m1, and 18S 4310893E) in 7900 HT Fast Real-Time PCR System (Thermo Fisher Scientific). mRNA expression relative to housekeeping gene (18S) was determined by using the ΔΔCT method.

### Statistics.

All data are shown as mean ± SEM. Analysis was by either 1-way or 2-way ANOVA (as appropriate) with post hoc Holm-Šídák test or by 2-tailed *t* test (GraphPad Prism 8.0). Significance was set at *P* < 0.05 for all analyses.

### Study approval.

All animal procedures were approved under the British Home Office Animals (Scientific Procedures) Act 1986 (Project Licence 70/7229 from Home Office, London, United Kingdom).

## Author contributions

JG conceived the study; YM, RR, IDB, CIE, MSNT, JC, BJ, CDJ, BYHL, GAR, and GSHY investigated; JG, YM, RR, and WSD wrote the original draft; all authors wrote, reviewed, and edited the manuscript. JG, GAR, and WSD supervised and acquired funding.

## Supplementary Material

Supplemental data

## Version 1 03/31/2020

In-Press Preview

## Version 2 04/23/2020

Electronic publication
