# Supplementary material for: Glucose in the hypothalamic paraventricular nucleus regulates GLP-1 release
Source: JCI Insight. 2020 Mar 31;5(8):e132760. doi: 10.1172/jci.insight.132760 (PMC7205434; doi:10.1172/jci.insight.132760)
Supplement: Supplemental data [file jciinsight-5-132760-s019.pdf]

Supplementary Information

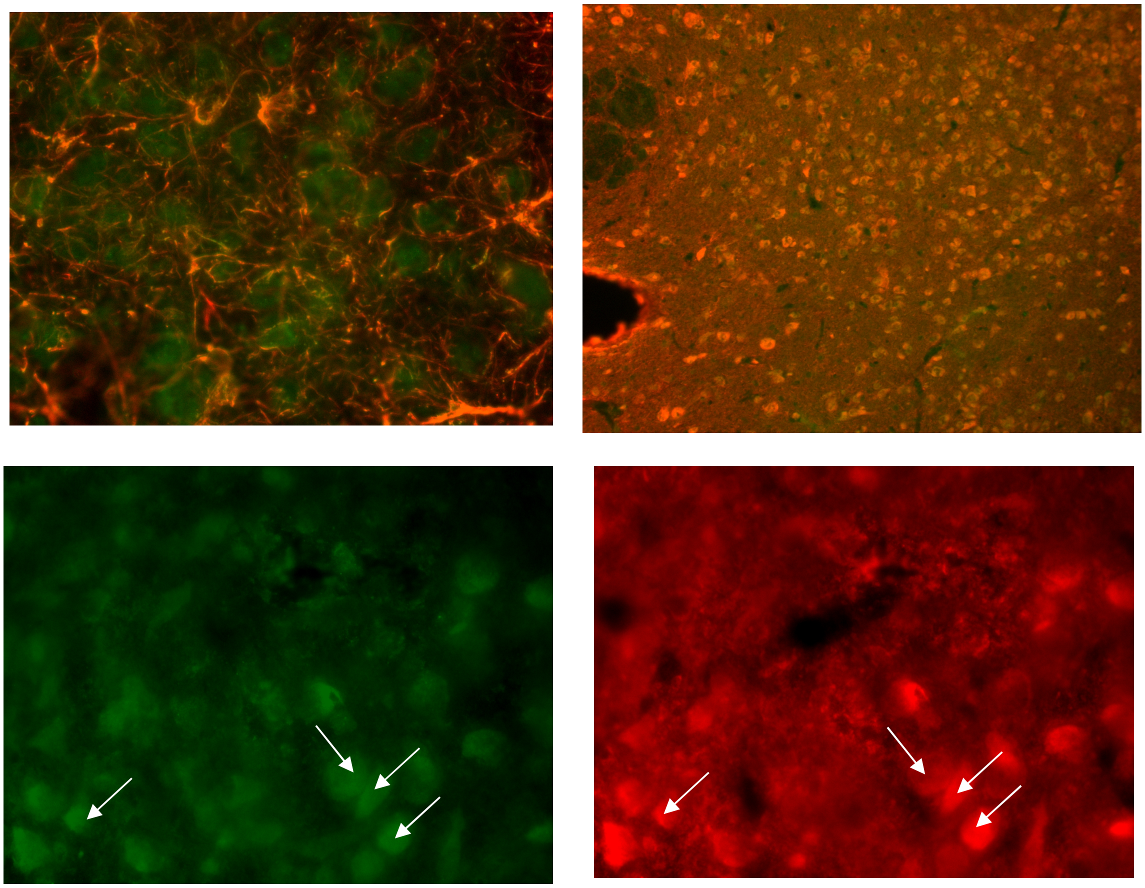

Figure S1: Immunohistochemically localisation of GFP following rAAV injection into the PVN.
(A) Immunohistochemical detection of both GFP (green) and GFAP (red) following injection of rAAV encoding GFP into the PVN. There is clear separation of the GFP and GFAP with no overlap of expression of the two magnificationx50
(B) Immunohistochemical detection of both GFP (green) and neurone specific PGP9.5 (red) following injection of rAAV encoding GFP into the PVN. Showing clear co-expression of the two proteins magnification x 20
(C) Immunohistochemical detection of GFP (green) following injection of rAAV encoding GFP into the PVN arrows indicate some cells which also express PGP9.5 magnification x50
(D) Immunohistochemical detection of neurone specific PGP9.5 (red) following injection of rAAV encoding GFP into the PVN arrows indicate some cells which also express GFP magnification x 50

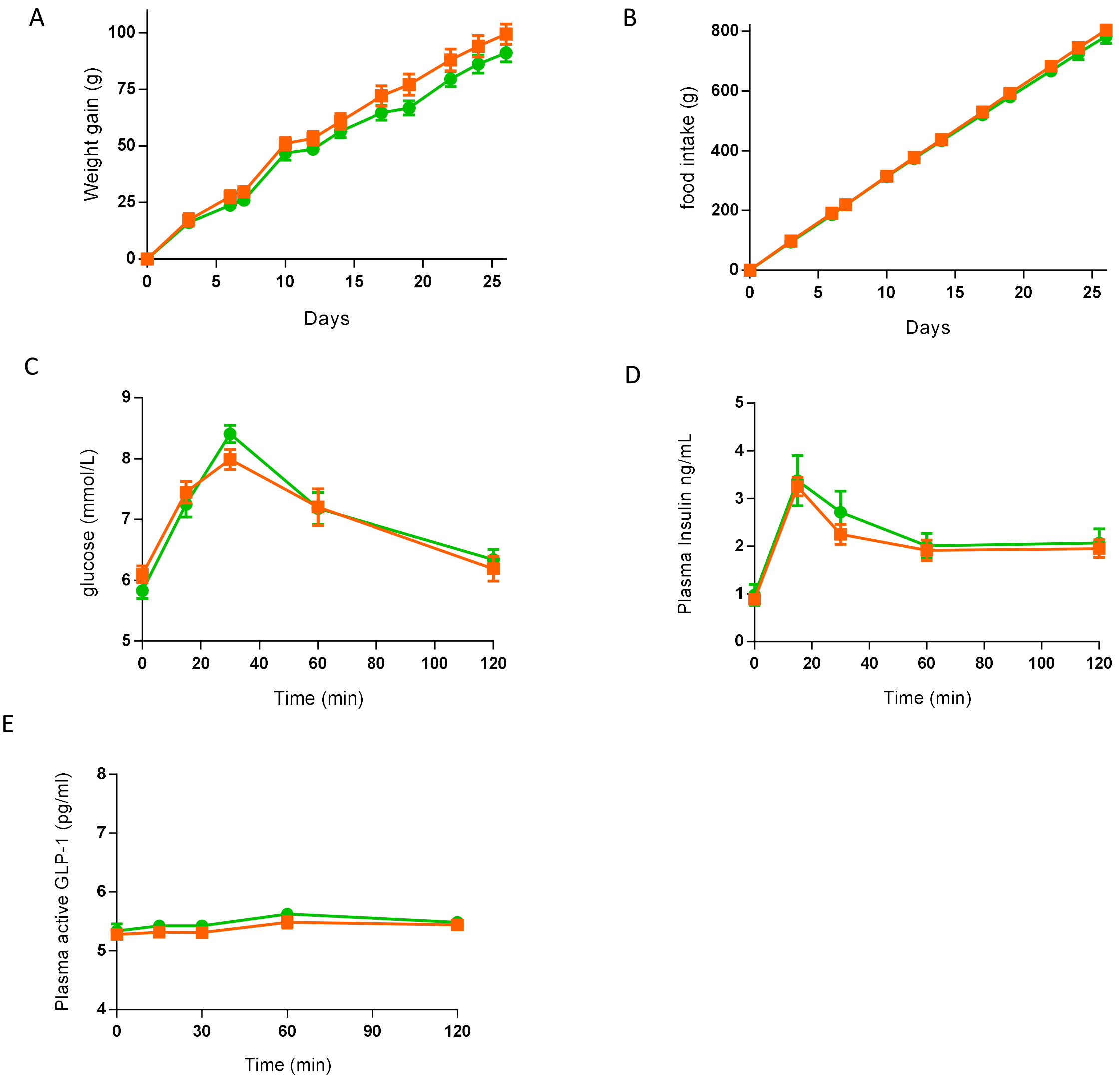

Figure S2: Effect of genetically increased paraventricular nucleus glucokinase activity on body weight, food intake, glucose intake and during an intraperitoneal glucose tolerance test.
Groups of adult male Wistar rats were injected with either rAAV expressing GFP (iPVN-GFP, green circles) or rAAV expressing full length glucokinase (iPVN-GK orange squares) bilaterally into the paraventricular nucleus.
(A) Body weight gain of iPVN-GFP (green circles) and of iPVN-GK (orange squares) rats.
(B) Cumulative food intake of iPVN-GFP (green circles) and of iPVN-GK (orange squares) rats
(C) Plasma glucose during an intraperitoneal glucose tolerance test in iPVN-GFP (green circles) and of iPVN-GK (orange squares) rats
(D) Plasma insulin during an intraperitoneal glucose tolerance test in iPVN-GFP (green circles) and of iPVN-GK (orange squares) rats
(E) Plasma active GLP-1 during an intraperitoneal glucose tolerance test in iPVN-GFP (green circles) and of iPVN-GK (orange squares) rats
Data are mean ± SEM, n=9-10. Data for A and B were analysed by GEE, for C, D and E were analysed by two way ANOVA and post-hoc Holm-Sidak’s.

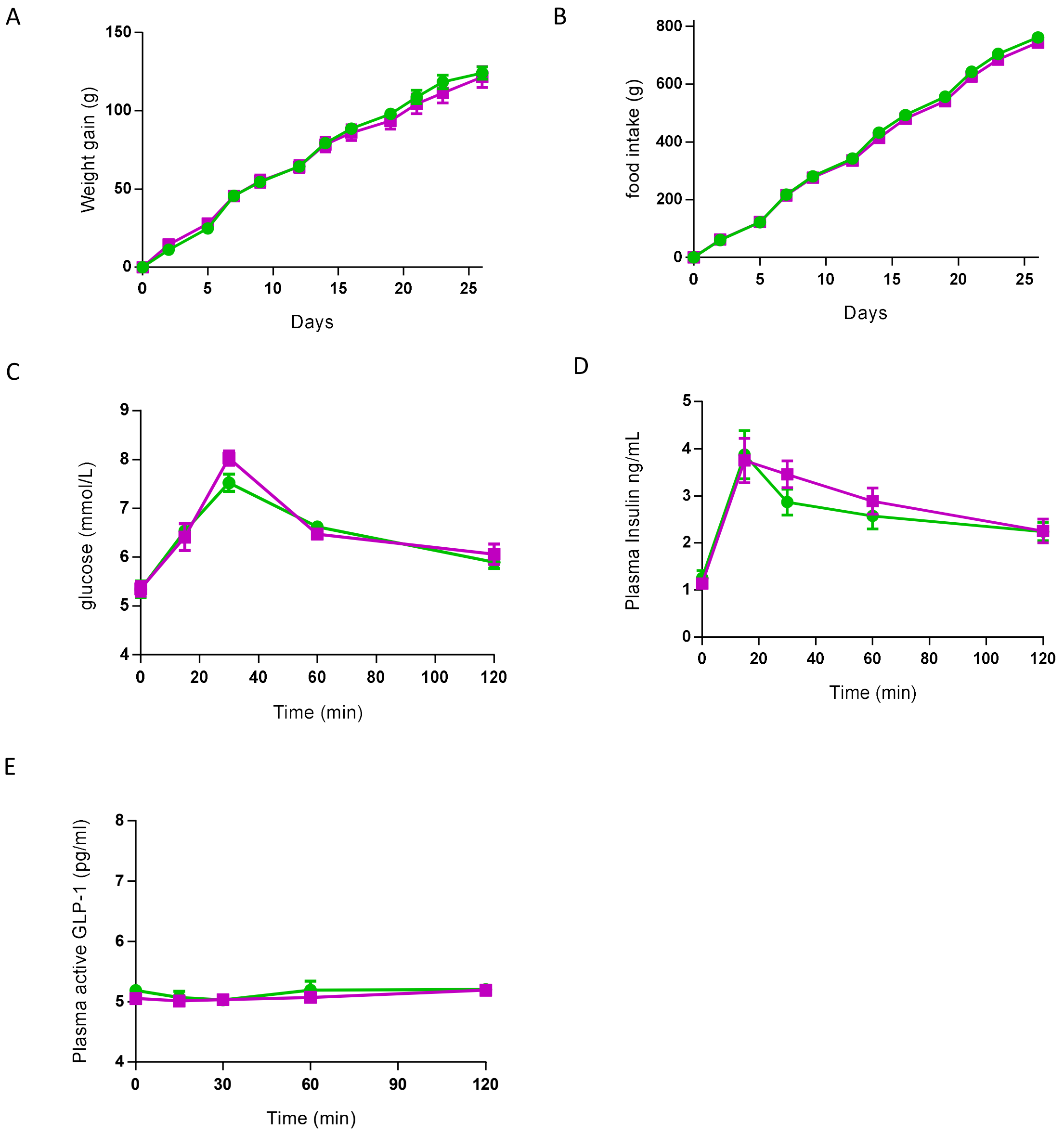

Figure S3: Effect of genetically decreased paraventricular nucleus glucokinase activity on body weight, food intake, glucose intake and during an intraperitoneal glucose tolerance test.
Groups of adult male Wistar rats were injected with either rAAV expressing GFP (iPVN-GFP, green circles) or rAAV expressing an antisense glucokinase construct (iPVN-ASGK magenta squares) bilaterally into the paraventricular nucleus.
(A) Body weight gain of iPVN-GFP (green circles) and of iPVN-ASGK (magenta squares) rats.
(B) Cumulative food intake of iPVN-GFP (green circles) and of iPVN-ASGK (magenta squares) rats
(C) Plasma glucose during an intraperitoneal glucose tolerance test in iPVN-GFP (green circles) and of iPVN-ASGK (magenta squares) rats
(D) Plasma insulin during an intraperitoneal glucose tolerance test in iPVN-GFP (green circles) and of iPVN-ASGK (magenta squares) rats
(E) Plasma active GLP-1 during an intraperitoneal glucose tolerance test in iPVN-GFP (green circles) and of iPVN-ASGK (magenta squares) rats
Data are mean ± SEM, n=8. Data for A and B were analysed by GEE, for C, D and E were analysed by two way ANOVA and post-hoc Holm-Sidak's.
